# Overexpression of Mena is associated with tumor progression and poor prognosis in oral squamous cell carcinoma *via* EMT

**DOI:** 10.3389/fonc.2022.1052375

**Published:** 2022-12-23

**Authors:** Sijia Na, Hao Cui, Zhichen Guo, Xiang Liang, Karim Ahmed Sakran, Xiaomei Guo, Xingqiang Li, Linyang Xie, Yifei Zhu, Hong Qi, Junbo Tu

**Affiliations:** ^1^ Key Laboratory of Shaanxi Province for Craniofacial Precision Medicine Research, College of Stomatology, Xi’an Jiaotong University, Xi’an, Shaanxi, China; ^2^ Department of Oral and Maxillofacial Surgery, College of Stomatology, Xi’an Jiaotong University, Xi’an, Shaanxi, China; ^3^ State Key Laboratory of Oral Diseases and National Clinical Research Center for Oral Diseases, West China Hospital of Stomatology, Sichuan University, Chengdu, Sichuan, China; ^4^ Department of Oral and Maxillofacial Surgery, West China Hospital of Stomatology, Sichuan University, Chengdu, Sichuan, China; ^5^ Department of Pathology, College of Stomatology, Xi’an Jiaotong University, Xi’an, Shaanxi, China

**Keywords:** mena, oral squamous cell carcinoma, clinicopathological significance, prognosis, epithelial-mesenchymal transition

## Abstract

**Background:**

Mena, a cytoskeletal regulatory protein, is involved in actin-based regulation of cell motility and adhesion, and contributes to tumor invasion and metastasis. However, the role of Mena in oral squamous cell carcinoma remains unclear. This is the first research focusing on the prognostic value of Mena in OSCC. In this study, we aimed to investigate the correlation between Mena expression and clinicopathological significance, as well as prognostic value in OSCC.

**Methods:**

Mena gene expression profiles of OSCC and normal tissues were collected from Oncomine, TCGA, and GEO databases. Biological function was analyzed through GO, KEGG and GSEA enrichment. Further, the expression level of Mena and tumor-related markers in 151 OSCC specimens was examined by IHC staining based on tissue microarray. Kaplan-Meier analysis was used to assess the prognostic performance of Mena in OSCC.

**Result:**

Mena was generally upregulation in various malignancies, especially OSCC. The functional analyses indicated that Mena was involved in the assembly and regulation of actin, cell movement, and EMT. IHC staining revealed that high expression of Mena in OSCC was correlated with Lymphatic metastasis, TNM stage, E-cadherin, Vimentin, and MMP-2, but insignificantly Ki67. Kaplan-Meier analysis demonstrated that elevated expression of Mena was significantly associated with poor overall survival and disease-free survival of OSCC patients.

**Conclusion:**

Mena could be a novel biomarker for predicting the prognosis of OSCC patients, which supports a theoretical basis for developing molecular target therapy.

## 1 Introduction

Oral squamous cell carcinoma (OSCC) is the most common histological type of cancer in the oral cavity, accounting for approximately 90% of oral cancer which is ranked the 15th leading cause of mortality and 16th most common malignancy worldwide (1, 2). Unfortunately, the estimated incidence of cases and the anticipated deaths attributable to OSCC in 2018 in America are 33,950 and 6,800 respectively (3). Despite substantial advances in diagnosis and treatment, OSCC still maintains a 5-year survival rate of 50% (4, 5). Local invasion and lymph node metastasis are the main factors of poor prognosis and death in patients with OSCC (6). Therefore, early prevention and detection is a vital component in the control of cancer. Highly invasive OSCC will be predicted by detecting its invasion and metastasis molecular biomarkers, which is helpful in preventing tumor progression and obtaining a better prognosis (7).

Mammalian-enabled (Mena) protein is a member of the Ena/VASP family, and encoded by the mammalian homolog gene ENAH (Enabled homolog) of Drosophila Ena on chromosome 1, and is associated with the formation of invasive membrane protruding (8). Mena contains 4 exons named +, ++, +++ and 11a (8–10). In the process of tumor progression, alternative splicing of Mena produced a variety of functional isoforms. Mena expressed in invasive tumor cells contained +++ exons but lacked 11a exons, called Mena^INV^. Mena expressed in non-invasive tumor cells lack +++ exons but express an 11a exons, called Mena^11a^ (10). Isoforms that lack both 11a and +++ exons called Mena (11). Besides, another Splice Variant of Mena lacking internal exon 6 (MenaDv6) promotes invasive behavior in cancer cells (12).

As a cytoskeletal regulatory protein, Mena is also involved in the assembly and regulation of the cytoplasmic actin network and plays a role in actin-based regulation of cell motility and adhesion, which frequently contributes to tumor invasion and metastasis (13–18). Several studies have reported outcomes of differential expression of Mena protein in various cancers (19–21). In breast cancer, overexpression of Mena promotes the proliferation and invasion of tumor cells, but deletion of Mena inhibits the metastasis of breast cancer and upregulated Mena increases drug resistance to breast cancer (22, 23). Besides, elevated Mena is correlated with the tumor grade and stage of hepatocellular carcinoma (HCC), and high expression of Mena in HCC enhances the expression level of epithelial-mesenchymal transition (EMT) markers and improves the carcinogenicity of HCC (24, 25).

Although Mena protein is associated with a variety of cancers, the clinicopathological significance and prognostic value of Mena in OSCC remains unclear. Thus, the current study firstly sought to investigate the expression and clinical significance of Mena in OSCC, and its biological function. Besides, we also evaluated the prognostic value of Mena in patients with OSCC as per their clinicopathological factors.

## 2 Materials and methods

### 2.1 Bioinformatics analysis

#### 2.1.1 Data collection

Mena expression levels in different types of cancer and normal tissues were compared by Oncomine (26, 27) (https://www.oncomine.org/resource/login.html) online analysis database. The gene expression profiles of OSCC samples and normal tissues were collected from The Cancer Genome Atlas (TCGA, https://portal.gdc.cancer.gov/), and Gene Expression Omnibus (GEO) database (GSE3524 (28), GSE74530 (29), GSE138206, http://www.ncbi.nlm.nih.gov/geo/).

#### 2.1.2 ENAH differential expression analysis

The gene expression microarray data were processed by R software (version 4.0.4, https://www.r-project.org/), and the R package was used to convert probe symbols into gene symbols. The “Limma” package was used to identify differentially expressed genes between oral squamous cell carcinoma tissues and normal tissues. (Adjust *P* < 0.05 and absolute log_2_fold change > 1 were considered statistically significant). TIMER (https://cistrome.shinyapps.io/timer/) online database was used to analyze the differential expression of ENAH (encoding Mena protein) between tumor and adjacent normal tissues in all TCGA tumors studied by the Gene_DE module. GEPIA (http://gepia.cancer-pku.cn/index.html) database was used to compare the expression of ENAH in HNSC tumors and normal tissues. (*P*< 0.01 and absolute log_2_fold change > 1 were considered statistically significant).

#### 2.1.3 Protein-protein interaction network and gene set enrichment analysis

In addition, STRING (http://string-db.org) online database was used to construct the Mena-related functional genes visualized as interactive networks, and gene set enrichment analysis (GSEA) was used to evaluate the gene biological function between high ENAH expression group and low ENAH expression group. Hallmark Gene sets were selected for enrichment analysis. (Genomes with the thresholds of FDR q <0.05 and NOM *P*<0.05 were considered statistically significant).

### 2.2 The clinicopathological characteristics of Mena

#### 2.2.1 Collection of clinical specimens and information

151 human OSCC specimens were obtained from the department of pathology, the Stomatological Hospital of Xi’an Jiaotong University from May 2013 to June 2020. All 151 patients with OSCC agreed and signed the informed consent. The research plan and purpose, for the collection of clinical information of OSCC patients, and the use of archived pathological specimens, have been approved by the Medical Ethics Committee of the Stomatological Hospital of Xi ‘an Jiaotong University (xjkqll [2022] NO.028). The screening criteria were as follows:(1) Patients were pathologically diagnosed as oral squamous cell carcinoma; (2) Patients had received surgical resection; (3) Patients have complete clinical data and follow-up data; (4) The patient does not have other malignant tumors. Clinical data of the patients were obtained from the Department of Pathology, Hospital of Stomatology, Xi’an Jiaotong University, including age, gender, tumor recurrence, tumor grade, clinical or pathological stage of tumor (cT/pT) and lymphatic status (cN/pN). Follow-up data were obtained by telephone or medical records review. The patient sample was comprised of 93 males and 58 females, with a mean age of 62 years (ranging from 27 to 85 years). Due to out-of-control situations, the postoperative follow-up was available only for 85 patients. In context, Kaplan-Meier curves were applied to analyze the overall survival and disease-free survival of those 85 patients, (43 patients with high expression of Mena and 42 ones with low expression of Mena).

#### 2.2.2 Preparation of tissue microarray

All pathological paraffin blocks were stained with HE and checked diagnosis again. Select the effective area and mark it. According to the mark position, punch a 1.00mm diameter tissue core and placed it consecutively on the recipient blocks of approximately 2cm×2cm to make the tissue microarray (TMA) paraffin block.

#### 2.2.3 Immunohistochemical analysis

Immunohistochemistry (IHC) staining was performed using Leica Bond MAX automatic immunostaining machine (Leica Microsystems, Wetzlar, Germany). The tissue sections were deparaffinized and rehydrated with ethanol and heat-induced epitope retrieval at pH 9.0 for 20 minutes. Rabbit polyclonal anti-Mena (ab244417, Abcam, Cambridge, UK, 1:100), anti-E-cadherin (MAB-0738, Maixin, Fuzhou, CHN), anti-Vimentin (MAB-0735, Maixin, Fuzhou, CHN), anti-CD34 (Kit-0004, Maixin, Fuzhou, CHN), anti- MMP-2 (ab37150, Abcam, Cambridge, UK, 1:500), and Ki67 (MAB-0672, Maixin, Fuzhou, CHN) were used for IHC staining. Automatic staining was performed using a Bond Polymer Refine -HRP detection system (DS9800, Leica Microsystems, Wetzlar, Germany). It was developed with diaminobenzidine (DAB) and counterstained with hematoxylin. Images were analyzed by IHC profiler (30) of Image J for quantitative evaluation and automatic scoring. The software quantitatively determined the score as negative, low positive, positive, or high positive. Clinical and histopathological characteristics are shown in **Table 1**.

**Table 1:** The correlation between Mena expression and clinicopathological characteristics in OSCC.

Characteristics	Patients		Mena			
	n	%	High	Low	χ2	*P*
No. of patients	151	100.00	105	46		
Gender						
Male	93	61.59	66	27	0.234	0.628
female	58	38.41	39	19		
Age						
>60 years	93	61.59	68	25	1.466	0.226
≤60 years	58	38.41	37	21		
Tumor recurrence						
Yes	51	33.77	34	17	0.299	0.584
No	100	66.23	71	29		
Lymphatic metastasis						
Yes	87	57.62	68	19	7.208	**0.007**
No	64	42.38	37	27		
Differentiation						
Well	104	68.87	74	30	0.415	0.813
moderate	38	25.17	25	13		
poorly	9	5.96	6	3		
TNM stage						
I/II	48	31.79	25	23	10.119	**0.001**
III/IV	103	68.21	80	23		
E-cadherin						
High	56	37.09	33	23	4.728	**0.030**
Low	95	62.91	72	23		
Vimentin						
High	57	37.75	47	10	7.215	**0.007**
Low	94	62.25	58	36		
MMP2						
High	115	76.16	87	28	8.517	**0.004**
Low	36	23.84	18	18		
Ki67						
high	73	48.34	53	20	0.627	0.428
low	78	51.66	52	26		

The bold values mean that P-values < 0.05.

### 2.3 Statistical analysis

SPSS Statistics software 24.0 (IBM, Armonk, NY, United States) was used for all statistical analyses. Descriptive statistics were reported as mean or frequency and rate. The correlations of Mena with the clinicopathological factors and tumor-related markers were performed using the χ2 test. Kaplan-Meier method was used to plot the survival curve and compared by log-rank test. Cox regression analysis was used to determine the independent prognostic factor. The *P<0.05, **P<0.01 and ***P<0.001 represent statistical significance.

## 3 Results

### 3.1 The mRNA expression levels of mena in tumor and normal tissues

**Figure 1:**
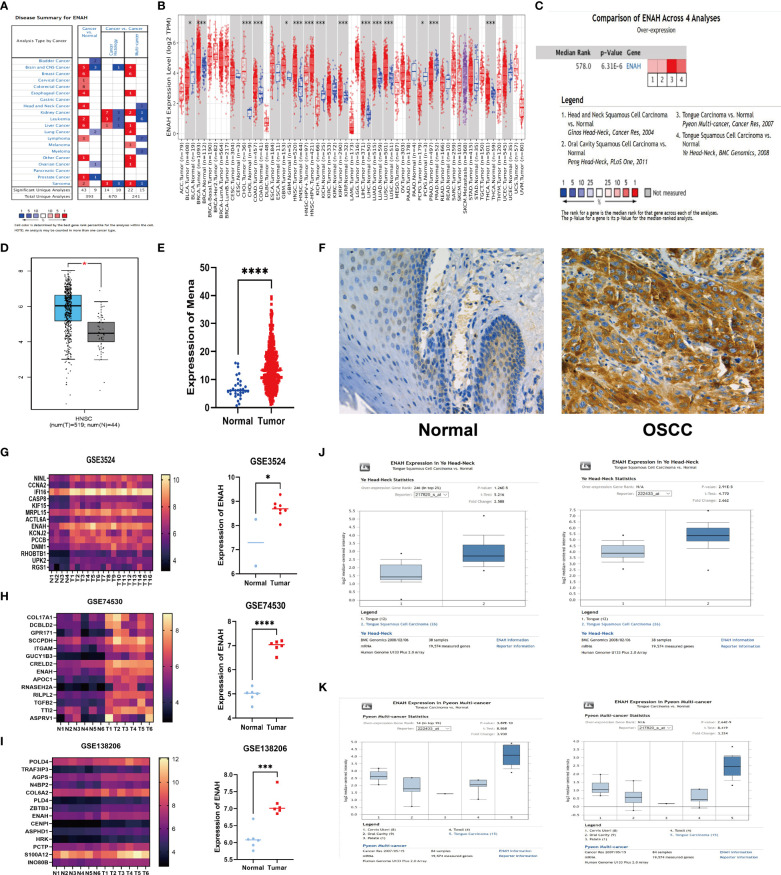
The abnormal expression of Mena mRNA between OSCC and normal tissues. **(A)** The expression of Mena mRNA in malignancies and normal tissues based on Oncomine database. **(B)** TIMER platform represents the expression of Mena mRNA in malignancies and normal tissues based on TCGA database. **(C, D)** Higher expression of ENAH (coded Mena) in HNSC tissues than normal tissues. **(E)** Mena expression between OSCC and normal specimens. **(F)** IHC staining shows lower or no expression of Mena in normal oral tissues. **(G-K)** The expression of Mena mRNA in OSCC and normal tissues based on GEO and Oncomine databases. *P < 0.05, ***P < 0.001, ****P < 0.0001 is mentioned in 2.3 statistical analysis.

In order to explore Mena mRNA levels in different malignancies and corresponding normal tissues, we analyzed 393 studies using the Oncomine database. 43 studies showed higher Mena expression in the brain and central nervous system, breast, cervix, colorectum, esophagus, head and neck, liver, prostate, leukemia, lymphoma sarcoma, and other malignancies, compared to normal tissues (**Figure 1A**). Consistent with the above result, we compared RNA sequencing data from various malignancies in the TCGA database using the TIMER platform. Mena expression was found to be higher in most tumor tissues (including HNSC) than in normal tissues (**Figure 1B**).

Notably, Mena expression was significantly elevated in head and neck squamous carcinoma (HNSC) tissues compared with normal tissues in 4 studies of HNSC (**Figure 1C**). Similarly, the comparison of 519 tumors and 44 normal tissues of HNSC in TCGA and GTEx databases by Gene expression Profiling Interactive Analysis (GEPIA) showed that ENAH (Mena encoding gene) in tumor tissues was 1.555-fold higher than that in normal tissues, which were 64.960 in tumor tissues and 21.444 in normal tissues (*P*<2.33E-10) (**Figure 1D**).

To further explore the expression of Mena in OSCC, we analyzed RNA‐seq data of 399 TCGA patients with OSCC (tumor 369, normal 30) which was then used to identify differentially expressed genes (DEGs) and found that Mena was higher in tumor tissues (Average 7.232 in normal vs. 14.311 in tumor; *P*<0.0001) (**Figure 1E**). Meanwhile, the analysis of 3 GEO datasets related to OSCC (GSE3524 GSE74530 GSE138206) showed that the expression level of ENAH in tumor tissues was significantly higher than that in normal tissues (In GSE3524, average 7.288 in normal vs. 8.70 in OSCC, *P*=0.0162; In GSE74530, average 4.97 in normal vs. 6.98 in OSCC, *P*<0.0001; In GSE138206, average 6.11 in normal vs. 7.12 in OSCC, *P*=0.0003) (**Figures 1G-I**). Subsequently, we performed IHC staining on OSCC tissues and normal oral tissues. Weak or no staining of Mena was observed in epithelial cells of normal oral tissues and keratin pearls with severe keratosis (**Figure 1F**). The same results were verified in two OSCC Oncomine datasets (Ye and Pyeon) (**Figures 1J, K**).

### 3.2 The biological function of Mena in OSCC

**Figure 2:**
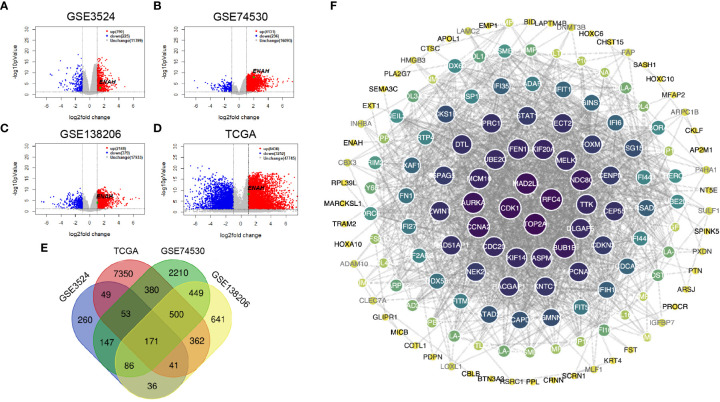
The construction and visualization PPI network of Mena based on DEGs from 3 GEO and 1 TCGA databases. **(A-D)** Volcano plots of DEGs from GSE3524, GSE74530, GSE138206, and OSCC-TCGA. **(E)** Venn diagram of 171 common DEGs in 3 GEO and 1 TCGA. **(F)** PPI network visualized by Cytoscape.

DEGs of GSE3524, GSE74530, GSE138206, and 399 OSCC specimens of TCGA were analyzed by Limma package. The volcano plots showing significant DEGs were shown in **Figures 2A–D**. The Venn diagram showed a total of 171 common DEGs from 4 different datasets (**Figure 2E**). The data indicated that Mena was all in the up-regulated group of DEGs. The PPI of DEGs was constructed by STRING with a confidence score>0.6, and the PPI network was visualized using Cytoscape. 171 nodes and 1034 edges were covered in the PPI network, which is worth noting that Mena is a member of the common DEGs (**Figure 2F**).

**Figure 3:**
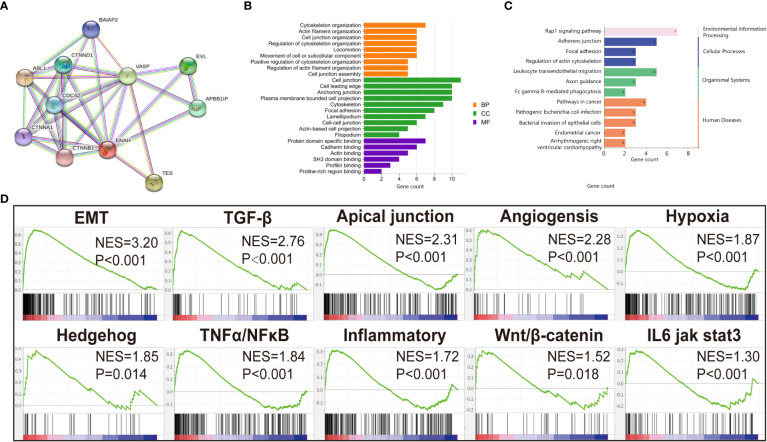
Functional annotation of Mena. **(A)** PPI network of Mena by STRING database. **(B)** Go enrichment analysis pf Mena related genes by STRING. **(C)** KEGG enrichment analysis of Mena related genes by STRING. **(D)** GSEA analysis to investigate the potential regulatory mechanisms with tumor hallmarks.

Since the differential expression of Mena was uncovered between OSCC and normal tissues, we next got an insight into the biological function of Mena in OSCC. By retrieving ENAH, we constructed a PPI network consisting of 11 nodes and 32 edges with a confidence score>0.7 utilizing the STRING database. The predicted functional genes associated with Mena mainly included ABL1, TES, VASP, EVL, APBB1IP, CTNND1, CDC42, BAIAP2, CTNNA1, and CTNNB1 (**Figure 3A**). Functional annotation including GO term and KEGG pathway analyses of these 11 Mena related genes were performed. GO annotation demonstrated that Mena-associated interacting proteins were mainly enriched in processes, such as “cytoskeleton organization”, “actin filament organization”, “cell junction organization”, “regulation of cytoskeleton organization”, “locomotion”, “movement of cell or subcellular component”, “positive regulation of cytoskeleton organization”, “regulation of actin filament organization”, “cell junction”, “cell leading edge”, “cytoskeleton, focal adhesion”, “lamellipodium”, “cell-cell junction”, “actin-based cell projection”, “filopodium”, “actin binding”, “profilin binding”, “proline-rich region binding” (**Figure 3B**). Meanwhile, KEGG pathway analysis indicated that the PPI network of Mena was significantly enriched in pathways like “pathways in cancer”, “endometrial cancer”, “focal adhesion”, “regulation of actin cytoskeleton”, “adherens junction”, “leukocyte transendothelial migration”, and “bacterial invasion of epithelial cells” (**Figure 3C**). The data of GO and KEGG annotation suggested that elevated expression of Mena may be involved in the cytoskeleton, regulation of actin assembly, and locomotion, which contributes to cell movement and migration, even tumor invasion and metastasis.

To further investigate the potential mechanisms of this hypothesis, 369 OSCC samples retrieved from TCGA database were divided into high Mena expression group and low expression group for gene set enrichment analysis (GSEA). We found that Hallmarks of tumor such as “epithelial-mesenchymal transition (EMT)”, “TGF-β pathway”, “Apical junction”, “Angiogenesis”, “Hypoxia”, “Hedgehog pathway”, “TNFα/NF-κB pathway”, “Inflammatory response”, “Wnt/β-catenin pathway”, and “IL6 jak stat3” were dynamically enriched in samples with high expression of Mena (**Figure 3D**). The result of GSEA analysis showed that Mena may promote tumor progression by participating in various cancer invasion-related signaling pathways, especially EMT and its related pathways.

### 3.3 The correlation between Mena and clinicopathological characteristics in OSCC

**Figure 4:**
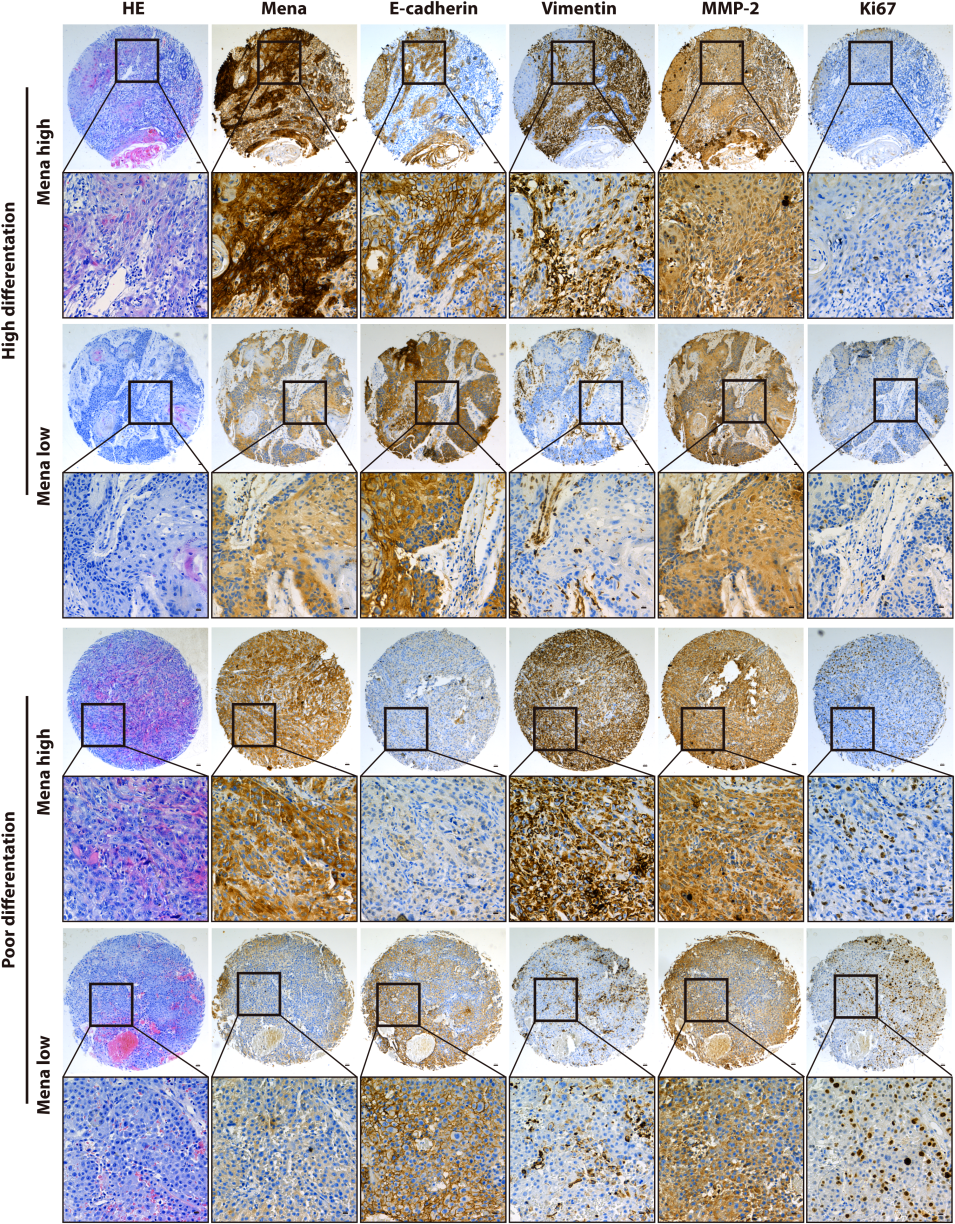
Immunohistochemical analysis of Mena protein and tumor-related markers expression.

Regarding Mena expression, there were 105 cases (69.5%) with high expression Mena, while 46 cases (30.5%) with low expression Mena (**Table 1**). The highest expression of Mena was predominantly present in the cytoplasm, followed by higher expression in the cell membrane than in the nucleus (**Figure 4**). These data showed that Mena was significantly increased in patients with OSCC.

In term of association with clinicopathological factors, Mena expression was significantly correlated with Lymphatic metastasis (χ2 = 7.208, *P*=0.007) and TNM stage (χ2 = 10.119, *P*=0.001) (**Table 1**). However, no significant correlation was found between Mena expression and other clinicopathological parameters, including Gender (χ2 = 0.234, *P*=0.628), Age (χ2 = 1.466, *P*=0.226), Tumor recurrence (χ2 = 0.299, *P*=0.584), and Differentiation (χ2 = 0.415, *P*=0.813) (**Table 1**).

The biological function and Mena expression in OSCC suggest that Mena expression may be closely associated with tumor invasion and metastasis. To prove this presumption, we analyzed the correlation between Mena and tumor-related markers. The results indicated that Mena expression was statistically associated with the expression of E-cadherin (χ2 = 4.728, *P*=0.03), Vimentin (χ2 = 7.215, *P*=0.007), and MMP-2 (χ2 = 8.517, *P*=0.004) but not Ki67 (χ2 = 0.627, *P*=0.428) (**Table 1**). Interestingly, Vimentin staining was stronger in most OSCC specimens with high-expression Mena, while E-cadherin staining was weaker. However, the staining results of Vimentin and E-cadherin were opposite in OSCC patients with low expression of Mena. Taken all, the evidences reveal that Mena may promote tumor progression through the EMT process.

### 3.4 Elevated expression of mena predicts the unfavorable prognosis of OSCC patients

**Figure 5:**
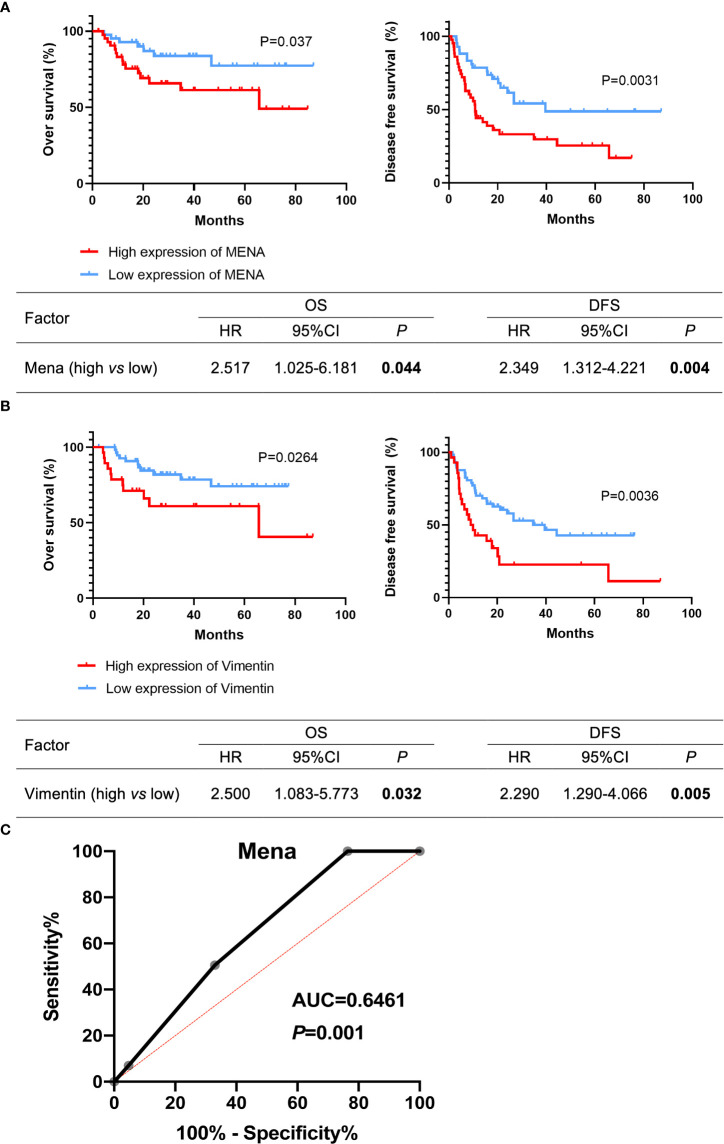
Potential prognostic value of Mena expression in OSCC patients. **(A, B)** Kaplan-Meier survival curves. **(C)** Receiver operating curves. The bold values mean that *P*-values < 0.05.

The prognostic value of Mena in OSCC patients was estimated by Kaplan-Meier survival analysis. OSCC patients with high Mena expression significantly shortened overall survival (OS) (*P*=0.037) and disease-free survival (DFS) (*P*=0.0031) (**Figure 5A**). Cox regression analysis revealed that elevated Mena expression was adverse prognostic factors for OS (HR 2.517, 95%CI 1.025-6.181; *P*=0.044) and DFS (HR 2.353, 95%CI 1.312-4.221; *P*=0.004) (**Figure 5A**). Increased Vimentin expression leaded to shorter OS (*P*=0.0264) and DFS (*P*=0.0036) in OSCC patients, and gave rise to worse OS (HR 2.500, 95%CI 1.083-5.773; *P*=0.032) and DFS (HR 2.290, 95%CI 1.290-4.066; *P*=0.005) (**Figure 5B**). The ROC curve of Mena presented diagnostic accuracy (area under the curve, 0.6461; *P*=0.001), compared with Vimentin (a typical EMT marker during metastasis progression) (**Figure 5C**). These results indicated that Mena played a negative role in the prognosis of OSCC patients, and it could be an optimal diagnostic biomarker for identifying OSCC invasion and metastasis.

## 4 Discussion

Oral squamous cell carcinoma remains a fatal and teratogenic disease. Tumor invasion and metastasis are the main factors of poor prognosis and death in patients with OSCC. Therefore, early prediction and treatment of invasive OSCC are of great significance to improve the prognosis of patients with OSCC.

Mena is a cytoskeletal regulatory protein, and it is first identified through its genetic interaction with Drosophila Abelson tyrosine kinase (D-Abl) (31, 32). Mena is located in the tips of filopodia, the lamellipodial leading edge, cell-cell contacts, and focal adhesions and actin stress fibers, and Mena protein is related to actin network remodeling and cell motility, and cytoskeleton regulation (8, 33–36). Cytoskeleton and actin dynamics of tumor cells play a critical role in this process of invasion and metastasis (37, 38). In the invasive tumor cells, Mena and other proteins (such as Arp2/3 complex (39, 40), Cofilin (41), and mDia2 (42) promote cytoskeleton and motor to drive the formation of membrane protrusions for invasion and motility (43, 44). Significantly increased Mena level has been revealed in a series of human cancers, such as breast cancer (45, 46), gastric cancer (47), hepatocellular carcinoma cells (28, 29), and esophageal carcinoma (48), highlighting the effect on promoting tumor cell proliferation and invasion. Nevertheless, there is a lack of studies on the relationship between Mena expression and oral squamous cell carcinoma.

In this study, bioinformatics analysis showed that in the four datasets (GSE3524, GSE74530, GSE138206, and 399 TCGA in OSCC patients), the expression of ENAH (the gene encoding MENA protein) in tumor tissues was significantly higher than that in normal tissues. The present results of immunohistochemical staining showed high expression of Mena in 105 (69.5%) OSCC patients. Further, elevated Mena expression was correlated with Lymphatic metastasis and TNM stage. Similar results have been reported in gastric cancer (47), cervical caner (20), and thyroid cancer (49). These results suggest that Mena increases the risk of tumor invasion and metastasis. The Mena PPI network was mainly enriched in the dynamic actin formation region, such as cell leading edge, cytoskeleton, focal adhesion, lamellipodium, actin-based cell projection, and filopodium. Their functions are related to the assembly and regulation of actin cytoskeleton and Locomotion, while Mena plays an important role in cell movement and migration through enhancing actin aggregation (13). As previously reported, hyperactivity of cell movement and migration leads to tumor invasion and metastasis and is involved in the progression of multiple cancers (50).

In order to further investigate the role of Mena in tumor invasion and progression, we performed biological function analysis by GSEA. The data showed that EMT, TGF-β signaling pathway, Apical junction, Angiogenesis, Hypoxia, etc. were enriched in high expression Mena phenotype. In the previous literature, TGF-β signaling pathway triggers the EMT process as an inducer (51), and the apical junction complex, including the tight junction and the adherens junction, plays a key role in tumor metastasis induced by EMT (52). E-cadherin, a vital apical junction complex, is a transmembrane protein responsible for anchoring neighboring cells to one another and forming adherens junctions. Decreased E-cadherin is necessary for the EMT process to occur, which promotes tumor metastasis (53). Additionally, the Mena intensity was associated with vascular invasion, and intensity of angiogenesis in colorectal carcinoma (20). Fibronectin (FN) levels are high around blood vessels and at invasive edges in tumors (54). Mena, as an actin regulatory protein, is an intrinsic mechanism driving tumor cells, which promotes haptotaxis along FN gradients (55). Thus, high expression levels of Mena^INV^ and FN are correlated with increased recurrence and poor prognosis in breast cancer. Hypoxia is a feature of solid tumors, and contributes to EMT, invasion and metastasis. Hypoxia regulated human Mena alternative splicing and promoted EMT through activating TGF-β-RBFOX2-ESRP1 axis in breast cancer (56). In addition, HIF-2α promoted glioma cells migration under hypoxia by activating an Oct-4/Sox-2-Mena^INV^ axis (57). Furthermore, our IHC results proved that high expression level of Mena was significantly correlated with EMT markers E-cadherin and Vimentin, and tumor invasive marker MMP-2, but uncorrelated with Ki67. In addition to E-cadherin, Vimentin, another key EMT marker widely expressed in mesenchymal and mesenchymal-derived cells, plays a role in maintaining cellular integrity and contributes to cell migration and invasion during EMT (58). Additionally, MMP-2 not only plays a key effect on ECM degradation in cancer progression, which contributes to cancer cell migration out of primary tumor to form metastases but also promotes EMT through activating TGF-β pathway (59, 60). As previously reported, EMT was initially described in the literature on normal cell differentiation in early development (61). In recent years, more and more studies found that EMT is the primary mechanism by which epithelial cancer cells acquire malignant phenotypes that promote metastasis (62). In pancreatic cancer, upregulation of Mena activates EMT induced by TGF-β1/Smad2 pathway (63). Overexpression of Mena in HCC has been reported to up-regulate the expression of EMT markers (24). All the above findings suggested that Mena may activate the invasion and metastasis of OSCC and accelerate the cancer progression by inducing the EMT process.

Survival analyses of OS and DFS uncovered that Mena is associated with poor prognosis in patients with OSCC. ROC curve analysis proved the diagnostic value of Mena for OSCC invasion and metastasis. The findings suggest that Mena may be helpful in decisions for therapeutic strategies. Further research will be conducted to investigate the molecular mechanism of Mena in the development and progression of OSCC.

In conclusion, this is the first study to reveal that increased expression of Mena in OSCC patients is associated with tumor invasion and metastasis resulting from EMT, as well as poor prognosis of OSCC. Therefore, Mena may serve as a novel biomarker to predict the prognosis of OSCC, which provides a theoretical basis for developing a molecular target therapy.

## Data availability statement

The original contributions presented in the study are included in the article/supplementary material, (Raw data included https://www.jianguoyun.com/c/sd/160b878/24df4ead91ec7249) further inquiries can be directed to the corresponding author/s.

## Ethics statement

This research has been reviewed and approved by the Medical Ethics Committee of the Stomatological Hospital of Xi ‘an Jiaotong University (xjkqll [2022] NO.028). The patients/participants provided their written informed consent to participate in this study.

## Author contributions

SN, HC, ZG, XL, and KS designed the study, analyzed data, and wrote the manuscript. JT and SN provided funding acquisition. XG, XQL, LX, and YZ performed the experiments and analyzed the data. JT, SN, and HQ supervised the research, analyzed data, and wrote the manuscript. All authors contributed to the article and approved the submitted version.
